# Navigating Dementia Care in India: An Analysis of Government Policies and Their Implications

**DOI:** 10.1002/alz70860_100314

**Published:** 2025-12-23

**Authors:** Vishnu Mangalamchery, Nalakath A. Uvais

**Affiliations:** ^1^ IQRAA International Hospital and Research Centre., Calicut, Kerala, India; ^2^ Iqraa International Hospital and Research Centre, Calicut, Kerala, India

## Abstract

**Background:**

Dementia poses a significant public health challenge in India. It ranks as the seventh leading cause of death and is a major contributor to disability and dependency among older adults. With an estimated 8.8 million individuals aged 60 and older living with dementia, the prevalence is expected to rise dramatically due to the aging population. Recognizing the urgent need for effective care, the Indian government has initiated dementia‐friendly policies aimed at enhancing access to affordable care services. This review study aims to analyze these government policies and their implications for dementia care in India.

**Method:**

This narrative review involved a comprehensive examination of national health policies, government reports, and peer‐reviewed literature to analyze dementia care policies in India. A targeted search was conducted using PubMed, relevant databases, and government websites to identify articles and reports related to dementia care, focusing on peer‐reviewed studies and policy documents that addressed frameworks and strategies for dementia care.

**Result:**

India has made notable progress in dementia care through initiatives such as the National Program for Health Care of the Elderly (NPHCE), which establishes geriatric services and memory clinics, and the Mental Healthcare Act (2017), which safeguards patient rights and promotes community‐based care. The National Health Protection Scheme (Ayushman Bharat) improves financial access to dementia care, while the District Mental Health Program (DMHP) integrates mental health services into primary healthcare for early detection and management. These policies collectively enhance awareness, train healthcare workers, and improve accessibility, contributing to a more inclusive and comprehensive dementia care framework.

**Conclusion:**

India's dementia care policies are moving in the right direction, with improved access to services and better training for healthcare providers. However, practical implementation remains a challenge, as available literature highlights gaps in diagnosis, treatment, and service delivery. Further research is needed to evaluate the on‐ground effectiveness of these policies and ensure they translate into equitable and comprehensive care for individuals with dementia.

